# Catechol-O-Methyltransferase Genotype, Frailty, and Gait Speed: The Cardiovascular Health Study

**DOI:** 10.1093/geroni/igaa057.2729

**Published:** 2020-12-16

**Authors:** Shannon Mance, Andrea Rosso, Joshua Bis, Stephanie Studenski, Nico Bohnen, Caterina Rosano

**Affiliations:** 1 University of Pittsburgh Graduate School of Public Health, Pittsburgh, Pennsylvania, United States; 2 School of Public Health, University of Pittsburgh, Pittsburgh, Pennsylvania, United States; 3 University of Washington, Seattle, Washington, United States; 4 University of Pittsburgh, Pittsburgh, Pennsylvania, United States; 5 University of Michigan, Ann Arbor, Michigan, United States

## Abstract

The association of COMT with gait speed varies across studies; frailty, a condition increasing vulnerability to stressors, may influence this association. Cross-sectional data was obtained in 3,744 participants (71 years, 82% white, 39% male) for gait speed, frailty (Fried definition), and COMT. Multivariable regression models of COMT predicting gait were adjusted for demographics, chronic conditions, and locomotor factors. Interactions of COMT by frailty and by race predicting gait speed were p=0.03 and p=0.02, respectively. Compared to Met/Met, the Val/Val group walked marginally more slowly in the full cohort (0.87 vs 0.89 m/sec, p=0.2); differences were significant for those with frailty (n=220, 0.55 vs 0.63m/sec, p=0.03), but not for those with moderate (n=1691, 0.81 vs 0.81m/sec, p=0.9), or no frailty (n=1833, 0.98 vs 0.97 m/sec, p=0.7). Associations were similar by race, but significant for whites only. Studies should assess the influence of dopaminergic signaling on gait slowing due to frailty.

